# Electric muscle stimulation attenuates neuroinflammation and improves the outcome of acute ischemic stroke in mice

**DOI:** 10.1016/j.neurot.2026.e00933

**Published:** 2026-06-02

**Authors:** Anna Törteli, Péter Kozák, Hiroyuki Uno, Rajmund Hősi, Zsófia Ruppert, Eszter Gáspár, Ferenc Bari, Zsolt Török, Eszter Farkas, Melinda E. Tóth, Ákos Menyhárt

**Affiliations:** a HCEMM-USZ Cerebral Blood Flow and Metabolism Research Group, HCEMM Nonprofit Ltd., Szeged, Hungary; b Department of Cell Biology and Molecular Medicine, Albert Szent-Györgyi Medical School and Faculty of Science and Informatics, University of Szeged, Szeged, Hungary; c HUN-REN Biological Research Centre, Centre of Excellence of the European Union, Institute of Biochemistry, Laboratory of Molecular Stress Biology, Szeged, Hungary; d Department of Medical Physics and Informatics, Albert Szent-Györgyi Medical School and Faculty of Science and Informatics, University of Szeged, Szeged, Hungary; e HOMER ION Laboratory Co., Ltd., Shinsen-cho 17-2, Shibuya-ku, Tokyo, 150-0045, Japan

**Keywords:** Electrical muscle stimulation, Ischemic stroke, Neuroinflammation, Muscle–brain signaling, Lactate metabolism

## Abstract

Acute ischemic stroke is a major cause of death and disability, yet many patients cannot engage in early rehabilitation due to severe motor deficits. Resulting immobility accelerates muscle atrophy and systemic inflammation, highlighting muscle–brain interactions as potential therapeutic targets. Electrical muscle stimulation (EMS) provides a non-volitional means of activating skeletal muscle and may mimic key neuroprotective features of exercise. We tested whether hyperacute EMS modulates muscle-to-brain signaling to improve stroke outcomes. Transient middle cerebral artery occlusion was induced in male and female C57BL/6 mice, followed by daily neurological assessments and 4 Hz lower-limb EMS for three days. Myofiber morphology, infarct size, blood lactate, and muscle and brain gene expression were subsequently analyzed. EMS preserved myofiber size and reduced stress-response gene expression (Hsp25, Hspb8, Atf4) in skeletal muscle. In the brain, EMS decreased infarct volume, limited necrosis, and improved neurological function. Stroke-associated inflammation was attenuated, evidenced by reduced *Tnf*, *Nlrp3 and Aif1* expression. EMS elevated circulating lactate, while stroke groups showed increased expression of the monocarboxylate transporter Mct-1, supporting a lactate-dependent metabolic coupling mechanism. These findings identify hyperacute EMS as a feasible, noninvasive intervention that confers neuroprotective and anti-inflammatory benefits after stroke, potentially via lactate-mediated muscle-to-brain signaling. EMS may represent a valuable adjunct for patients unable to mobilize during the critical early phase of stroke recovery.

## Graphical abstract

**Figure:**
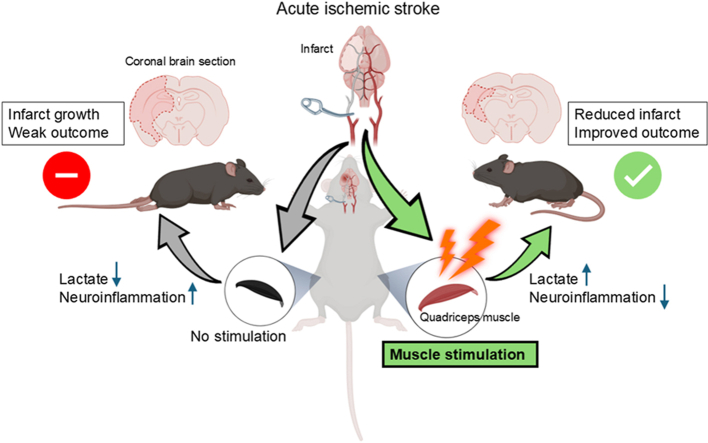

## Introduction

Acute ischemic stroke (stroke) remains a leading cause of death and long-term disability worldwide [1]. Although recanalization therapies, including intravenous thrombolysis and endovascular thrombectomy, have improved early outcomes, more than half of stroke survivors develop persistent neurological deficits and increased mortality during the subacute and chronic phases [1]. Despite extensive experimental efforts, effective neuroprotective strategies have not translated successfully into clinical practice, highlighting the need for alternative therapeutic approaches [2]. Current stroke guidelines emphasize early mobilization as a key determinant of functional recovery, particularly once treatment options are largely limited to rehabilitation only [3]. In fact, prolonged immobilization after stroke is associated with systemic muscle wasting affecting both paretic and non-paretic limbs and contributing substantially to long-term disability and inefficient stroke rehabilitation [4,5]. Also, immobilization was found to be associated by significant neurological decline of bedridden stroke patients [6]. Likewise, muscle wasting was shown to correlate with the severity of brain injury after ischemic strokes in mice [7]. These observations raise the possibility of a dysfunctional muscle→brain communication rooted in muscle atrophy, which - if intact - would be essential to assist neuroprotection and regeneration after stroke.

Early mobilization after stroke reduces immobility-related systemic inflammation, promotes perfusion-dependent neuroplasticity, and may attenuate neuroinflammation-a key contributor of infarct maturation-through humoral and sensory pathway activation [[8], [9], [10]]. However, a substantial proportion of patients are unable to participate adequately in early mobilization due to severe motor deficits, reduced consciousness, or medical complications [11]. In this context, electrical muscle stimulation (EMS) can be applied as an adjunct therapy, enabling skeletal muscle activation in immobilized patients and partially reproducing exercise-related systemic and neuroimmune effects [12,13]. Thus, EMS may augment early mobilization in patients unable to move voluntarily during the acute or early subacute phase; however, current evidence remains limited and provides little mechanistic insight [14].

Here we developed a novel, electric muscle stimulation-based intervention that is applicable to mice with post-stroke paralysis and/or restricted mobility. With our therapy we aimed to improve the outcome of stroke by confirming our hypothesis that the muscle-brain communication axis is essential for stroke recovery. We demonstrate for the first time that low-frequency, non-invasive hindlimb stimulation exerts anti-inflammatory effects in both skeletal muscle and the brain, while reducing infarct size and improving outcomes after stroke in mice.

## Materials and methods

### Animals

The study adhered to established ethical guidelines [15] and the experiments are reported in compliance with the ARRIVE guidelines [16]. The experimental procedures were conducted in strict accordance with the guidelines of the National Food Chain Safety and Animal Health Directorate of Csongrád County, Hungary, and the guidelines of the Scientific Committee of Animal Experimentation of the Hungarian Academy of Sciences (updated Law and Regulations on Animal Protection: 40/2013. (II. 14.) Gov. of Hungary), following the EU Directive 2010/63/EU on the protection of experimental animals (Ref. nr. I-74-23/2022. and II./761/2024. MÁB).

The animals were housed under constant conditions of temperature (23 °C), humidity, and lighting (12:12 h light/dark cycle, lights on at 7 a.m.). Standard rodent chow and tap water were supplied ad libitum. Adult female and male 3–7-month-old C57BL/6 mice (Charles River Laboratories; weighing 27 ± 3 g; *n* = 90, from the husbandry of the Biological Research Center, Szeged, Hungary) were used in this study. Mice were assigned to four experimental groups: Control (*n* = 16), electric muscle stimulation (EMS, *n* = 16), Stroke (*n* = 29), and Stroke + EMS (*n* = 29).

### Induction of acute ischemic stroke (stroke) in mice

Stroke was induced by transient occlusion of the left middle cerebral artery (MCAO) using the in-house modified Koizumi method to allow complete reperfusion as described earlier [15,17]. In brief, mice were placed supine, the neck area was disinfected, and 1% lidocaine was applied before a midline cervical incision. The left common, external, and internal carotid arteries were carefully exposed. A Micro Serrefine (Fine Science Tools (USA), Inc.) was placed on the common carotid artery, and a silk suture was tightened around the external carotid artery to temporarily block blood flow. A small incision was made in the common carotid artery, through which a 230-μm silicon-coated filament (Doccol Corp., Sharon, MA 02067–2427, USA) was advanced via the internal carotid artery to occlude the middle cerebral artery. MCAO was confirmed by laser Doppler flowmetry (Probe 403 connected to PeriFlux 5000; Perimed AB, Sweden) and only animals showing a cerebral blood flow reduction below 25% of baseline were included. After 60 min of occlusion, complete reperfusion was achieved by the withdrawal of the occluding microfilament, while the incision in the common carotid artery was gently sealed with a collagen tamponade (Gelita Tampon, B. Braun, Spain) to prevent bleeding. The external carotid ligature and clamp were removed, the wound was sutured and disinfected, and carprofen (5 mg/kg, s.c.) along with saline (1 ml) was administered. Animals recovered in a heated incubator before being returned to their home cages.

### Post-operative care of mice after stroke

Postoperative care included the provision of food, water, and nutritional supplements as described previously [15]. In brief, during the 24–72 h after surgery, mice received maximal nutritional support with ad libitum access to soaked pellets and jelly food (DietGel® Recovery, ClearH2O, INC. 85 Bradley Drive Westbrook, ME 04092). Additional nutrition was provided twice daily by syringe feeding. Fluid support consisted of twice-daily subcutaneous injections of 0.3 ml saline containing 5% glucose and a 50:50 Duphalyte (Zoetis Hungary Kft) solution. Carprofen was administered every 12 h.

### Characterization of neurological deficit

Animals underwent neurological testing 24 h prior to surgery and daily from 24 to 72 h after MCAO, in accordance with our previous observations [15]. Sensorimotor deficits were assessed using the Garcia Neuroscore Scale, a validated scoring system for ischemic brain injury in rodents. The GNS ranges from 0 (severe deficit) to 21 (no deficit). All test domains were performed in the same order for each animal, with three investigators present during testing to ensure unbiased scoring.

### TTC staining of brain infarcts

The size of brain infarcts was determined by triphenyltetrazolium chloride (TTC) staining. After the termination of the experiment, the brain was carefully sectioned to 1 mm thick coronal slices in a dedicated matrix, then the individual slices were incubated in a 2% TTC solution at 37 °C for 25 min. The infarct size was expressed as the % of the affected brain hemisphere.

### Hematoxylin-eosin staining of brain and muscle samples

Both coronal brain and musculus quadriceps sections were cut into 10-μm-thick frozen sections using a cryostat (Leica CM1860 UV, Leica, Germany). Sections were stained with hematoxylin and eosin (Sigma-Aldrich, USA) and coverslipped with Eukitt® (Merck, USA). Photomicrographs were acquired at 20 × magnification using a Nikon DS-Fi3 camera mounted on a Leica DM2000 LED light microscope (Leica Microsystems GmbH, Germany).

### Electric muscle stimulation (EMS) therapy

**Fig. 1:**
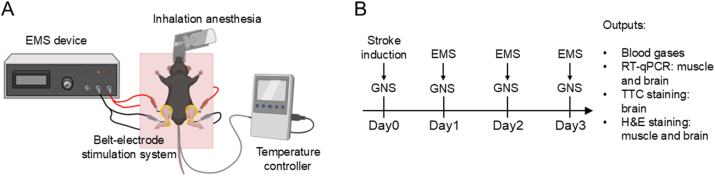
Illustration of the applied EMS therapy and experimental protocol. Panel A: Schematic drawings represent the Belt electrode Skeletal muscle Electrical Stimulation (B-SES) mounted on a mouse lying on supine position. The nose cone indicates the inhalation anesthesia (isoflurane) during the EMS therapy. The yellow belt electrodes are wrapped around the lower limb proximal thigh and ankles of the mouse. The electrodes are connected to the electric muscle stimulation device (EMS device) with red (cathode) and black (anode) cables. Body temperature is maintained at 37 °C using a thermostat-controlled heating pad. Panel B: Experimental protocol. The neurological scoring of mice (Garcia Neuroscore Scale, GNS) starts on Day0 preceding stroke induction and is continued through the subsequent 3 days (from Day1-Day3) following stroke induction on Day0. Black arrows indicate the electric muscle stimulations (EMS) started on Day1. EMS sessions were performed on 3 subsequent days (from Day1-Day3) after stroke. The experiments are terminated on Day3, where the indicated outputs: blood gases, RT-qPCR of muscle and brain samples, TTC staining of brain sections and H&E staining of muscle and brain samples were analyzed.

The EMS therapy applied here was based on the previously optimized Belt electrode Skeletal muscle Electrical Stimulation (B-SES; HOMER ION Laboratory Co., Ltd., Tokyo, Japan) and setup developed by HOMER ION Laboratory Co., Ltd. A great advantage of B-SES is that the entire belt surface functions as an electrode, enabling uniform electrical stimulation across the whole lower limb [18]. Moreover, because the current is distributed over a larger area, this approach minimizes discomfort upon muscle contractions. The protocol applied in our study was designed to mouse stroke experiments in collaboration with HOMER ION Laboratory Co., Ltd. First, mice were anesthetized with isoflurane (5% for induction, 0.6%–0.9% for maintenance in N_2_O:O_2_, 2:1) and allowed to breath spontaneously through a nose cone. Body temperature was maintained at 37 °C by using a heating pad equipped with a temperature probe and blanket feedback-controlled system (CODA Monitor, Kent Scientific Corporation) (Fig. 1A). The B-SES system consisted of a main control unit (for stimulation cycle, frequency and intensity setting) and the belt electrode pairs, wrapped around the lower limb proximal thigh and ankles of mice (Fig. 1A). The electrode-skin contact was ensured by (i) the hair removal and waxing of the lower limbs and (ii) the continuous application of physiological saline (0.9% NaCl) onto the skin surface. Then, EMS therapy (B-SES frequency, 4 Hz) was applied to the bilateral lower-limb skeletal muscles. The optimal stimulus intensity was personalized to each mouse; the intensity of EMS was set by the experimenter until the current achieved visible muscle contraction of the lower limb (see supplementary video). The first EMS session began on Day 1 and continued for three consecutive days after stroke. On Day 1, EMS was applied for 15 min, followed by a 15-min rest period, and then another 15-min EMS session. On Day 2, the duration of the second session was increased to 20 min, and on Day 3, both sessions were extended to 20 min each. Fluid supplementation (described above) was administered during each 15 min break. The whole protocol lasted for 60–65 min per day. Stroke mice served as controls for the EMS therapy and received the same dose and duration of isoflurane anesthesia. Similarly, mice in the Control and EMS groups were administered the same dose and duration of anesthesia.

Supplementary video related to this article can be found at https://doi.org/10.1016/j.neurot.2026.e00933

The following is the supplementary data related to this article:

Multimedia component 2

### Experimental protocol

Stroke was induced on Day 0 following baseline neurological assessment using the Garcia Neuroscore Scale. Animals were then allowed to recover for 24 h. On Days 1, 2, and 3, GNS scoring was performed and followed by an EMS session. Experiments were terminated on Day 3, at which time blood was collected via cardiac puncture, muscle samples (musculus quadriceps, musculus soleus, and musculus gastrocnemius) were harvested from the hind limbs, and brain tissue was collected for histological and gene expression analyses (Fig. 1B). Importantly, TTC staining of brain infarcts was performed on 10 Stroke and 10 Stroke + EMS mice, while H&E staining and brain PCR analyses were conducted in a separate cohort (5 mice per group). This independent validation across multiple experimental sets strengthens the robustness and relevance of our findings.

### RNA isolation and quantitative real-time polymerase chain reaction (qPCR)

Total RNA was isolated using RNA and protein purification kit (Macherey-Nagel, Düren, Germany) from brain samples and RNeasy Fibrous Tissue Mini Kit (Qiagen, Hilden, Germany) from muscle samples according to the manufacturer’s instructions. High-Capacity cDNA Reverse Transcription Kit (Thermo Fisher Scientific, Waltham, Massachusetts, USA) was used to convert RNA samples to cDNA. Each reaction mixture contained 1 μg RNA (15 μl), 1.5 μl MultiScribe Reverse Transcriptase, 3 μl primer, 1.2 μl dNTP, 3 μl buffer, 6.3 μl RNase-free water. Parameters for the reverse transcription program were the following: incubation at 25 °C for 10 min, reverse transcription at 37 °C for 2 h, and inactivation at 85 °C for 5 min (using BioRad T100 Thermal Cycler, Hercules, CA, USA). The cDNA product was finally diluted at 1:20 and used as a qPCR reaction template. For the qPCR reaction, 10 μl cDNA, 1 μl (250 nM final) primer mix (forward + reverse), and 10 μl Power SYBR Green PCR Master Mix 2x (Thermo Fisher Scientific, Waltham, Massachusetts, USA) were mixed in a total volume of 20 μl. The reaction was performed on a RotorGene 3000 instrument (Qiagen, Hilden, Germany) with the following settings: heat activation at 95 °C for 10 min followed by 40 cycles of denaturation at 95 °C for 15 s, annealing at 60 °C for 60 s. Melting curve analysis was performed between 50 and 95 °C to verify the specificity of the amplification. Primer sequences used in qPCR reactions are listed in Table 1. The mouse *Gapdh* (glyceraldehyde 3-phosphate dehydrogenasegene) gene served as an internal control for normalization. Relative gene expression levels were calculated using the ΔΔCt method.

**Table 1:** Primer sequences used for qPCR.

	gene	forward primer	reverse primer
**1**	***Gapdh***	GGGTTCCTATAAATACGGACTGC	CCATTTTGTCTACGGGACGA
**2**	***Tnf***	CCCTCACACTCAGATCATCTTCT	GCTACGACGTGGGCTACAG
**3**	***Il1b***	GCAACTGTTCCTGAACTCAACT	ATCTTTTGGGGTCCGTCAACT
**4**	***Tgfb1***	CTCCCGTGGCTTCTAGTGC	GCCTTAGTTTGGACAGGATCTG
**5**	***Il10***	CAGAGCCACATGCTCCTAGA	TGTCCAGCTGGTCCTTTGTT
**6**	***Il6***	GCTACCAAACTGGATATAATCAGGA	CCAGGTAGCTATGGTACTCCAGAA
**7**	***Nlrp3***	TACCCAAGGCTGCTATCTGGA	GCTTAGGTCCACACAGAAAGT
**8**	***Erfe***	TCCTCTATCTACAGGCAGGACA	GAAGTGAGAGCCACTGCGTA
**9**	***Fndc5***	TTGCCATCTCTCAGCAGAAGA	GGCCTGCACATGGACGATA
**10**	***Ctsb***	ATTCACACCAATGGCCGAGT	TAGCCACCATTACAGCCGTC
**11**	***P62***	GAACTCGCTATAAGTGCAGTGT	AGAGAAGCTATCAGAGAGGTGG
**12**	***Atg4***	GAAGGAAGTTTTCCCCGATTGG	GGGTTGTTCTTTTTGTCTCTCCC
**13**	***Lc3***	GACCGCTGTAAGGAGGTGC	CTTGACCAACTCGCTCATGTTA
**14**	***Xbp1s***	CTGAGTCCGAATCAGGTGCAG	GTCCATGGGAAGATGTTCTGG
**15**	***Atf4***	CTAAGCCATGGCGCTCTTCA	GCTGGATTCGAGGAATGTGC
**16**	***Chop***	CCACCACACCTGAAAGCAGAA	AGGTGAAAGGCAGGGACTCA
**17**	***Myh8***	GAAGTGATTCAAGAGTCACGCA	CCATCATGGCGGCATCAGTA
**18**	***Snat1***	CCCTTTATTTCTCGAGGGGTCT	CGCAGTCAAAAGTCAACCCA
**19**	***Cdkn1a***	GCAGAATAAAAGGTGCCACAGG	GACAACGGCACACTTTGCTC
**20**	***Hsp25***	ATCCCCTGAGGGCACACTTA	GGAATGGTGATCTCCGCTGAC
**21**	***Cryab***	GTTCTTCGGAGAGCACCTGTT	GAGAGTCCGGTGTCAATCCAG
**22**	***Hsp70***	GAGATCGACTCTCTGTTCGAGG	GCCCGTTGAAGAAGTCCTG
**23**	***Hspb8***	AGACCCCTTTCGGGACTCA	GGCTGTCAAGTCGTCTGGAA
**24**	***Hspb3***	AGACCCCAGTGCGTTATCAG	GCAGTGCGTATAGTGTATGATCC
**25**	***Gfap***	CGGAGACGCATCACCTCTG	AGGGAGTGGAGGAGTCATTCG
**23**	***Aif1***	ATCAACAAGCAATTCCTCGATGA	CAGCATTCGCTTCAAGGACATA
**27**	***Cd68***	GACCTACATCAGAGCCCGAGT	CGCCATGAATGTCCACTG
**28**	***Itgam***	CAATAGCCAGCCTCAGTGC	GAGCCCAGGGGAGAAGTG
**29**	***Mrc1***	CCACAGCATTGAGGAGTTTG	ACAGCTCATCATTTGGCTCA
**30**	***Arg1***	GAATCTGCATGGGCAACC	GAATCCTGGTACATCTGGGAAC

qPCR data are presented as % of the control group. Normal distribution was checked using the Shapiro–Wilk normality test. In the case of normal distribution, the parametric Student’s t-test was performed for pairwise comparisons. If the normal distribution test failed, the non-parametric Mann–Whitney *U* test was used for pairwise comparisons.

### Data analysis and statistics

Power analysis for Stroke + EMS vs. Stroke experiments followed established principles. The pilot experiments indicated differences between the experimental groups (Stroke + EMS vs. Stroke) and achieved the confidence level of 95% and a power of 80% at low sample size (*α* = 0.05 and β (type II error) of 0.2). As calculated, sufficient statistical power was assumed at a final sample size of 10–12 animals/group. The calculations were run in GPower 3.1 (Heinrich Heine University of Düsseldorf, Germany).

The mice were independently coded and randomized. Surgeries were performed by two investigators, while neuroscoring and histological analyses of brain and muscle samples were conducted by four blinded individuals. Neuronal necrosis was evaluated using the built-in automated “Analyze Particles” plug-in in Fiji. Cell numbers were consistently quantified in the cortical region of mice within a standardized 1000 μm^2^ square region of interest defined in Fiji. qPCR analyses were performed on double-blinded, coded samples by three independent collaborators. Data are presented as mean ± SD or as fold changes (%) in RT-qPCR samples (Fig. 4, Fig. 5). Parametric or non-parametric statistics were chosen based on the results of a Shapiro-Wilk test of normality performed on the data sets. The statistical analysis was conducted using SigmaPlot 12.5 (Systat Software, Inc.). The specific statistical methods used are described in detail in each figure legend.

**Fig. 2:**
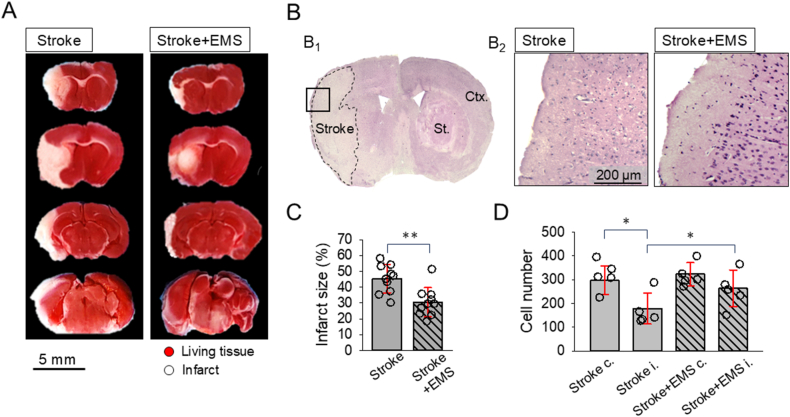
Electric muscle stimulation (EMS) therapy attenuates infarct size 3 days after stroke. Panel A: EMS therapy caused a significant reduction of infarct size as displayed by the representative TTC stained coronal sections and evaluation of dataset (Panel C). Panel B: Neuronal necrosis in hematoxylin-eosin (HE) stained sections. A representative H&E stained coronal brain section (B1). The infarcted tissue shows low H&E staining and is indicated by the black dashed line. The rectangle inlet shows the territory selected for cell counts on the 20x magnification H&E stained sections (B2). The top right (B2) images represent two inserts demonstrating the neuroprotective effect of EMS, as indicated by an increased number of purple dots (cells) and a reduced number of white-background cells. Panel D shows bar charts illustrating the significant effects of EMS. Abbreviations: TTC, triphenyl tetrazolium chloride; H&E, hematoxylin–eosin. Data are presented as mean ± SD. The distribution of data was evaluated by a Shapiro-Wilk test of normality (C, *p* = 0.937, D, *p* = 0.409), followed by a T-test (C) and a Two-Way ANOVA with Holm-Sidak post-hoc test (D), *p* < 0.01∗∗, *p* < 0.05∗.

**Fig. 3:**
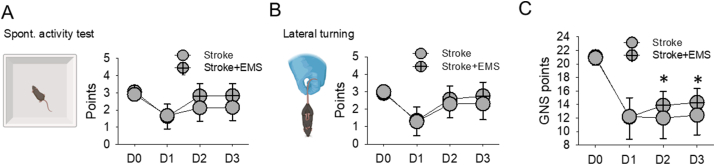
Electric muscle stimulation (EMS) therapy improves somatomotor functions of mice after stroke. Neurological deficit expressed on the Garcia Neuroscore scale (GNS) for subsequent 3 days after stroke with respect to pre-stroke performance (D0). Panel A: Spontaneous activity of mice was tested in an open field plexi cage (left cartoon) and was evaluated from D0-D3 (right diagram). Panel B: Lateral axial turning of mice when lifted by the tail (left cartoon and right diagram). Both Panel A and Panel B diagrams show only tendencies of changes. Panel C: GNS diagram shows significant difference on D2 and D3 between the experimental groups. Data are given as mean ± SD. The distribution of data was evaluated by a Shapiro-Wilk test of normality (C, *p* = 0.927), followed by a Two-Way ANOVA with Holm-Sidak post-hoc test, *p* < 0.05∗.

**Fig. 4:**
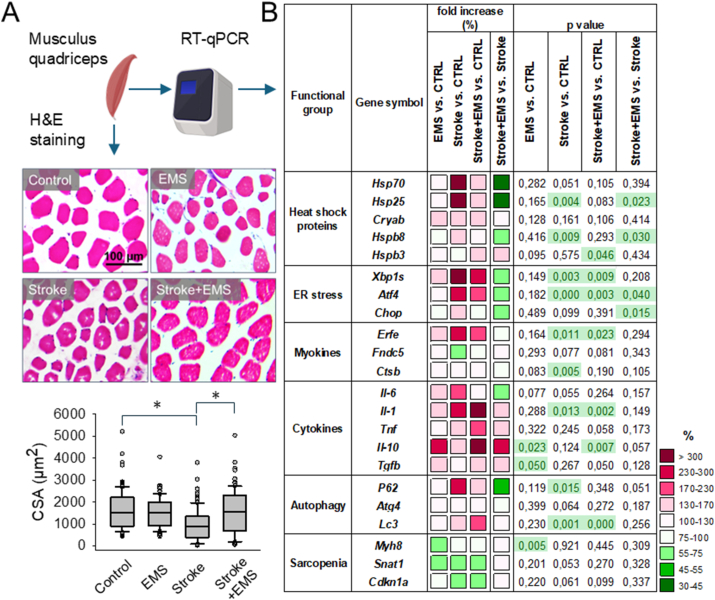
Histological evaluation and mRNA expression analysis of the left (ipsilateral) musculus quadriceps 3 days after stroke. Panel A: Cartoons show the bi-processing of m. quadriceps samples. Arrows point at the two directions of processing: hematoxylin-eosin (HE) staining and RT-qPCR analysis. Representative images display myofiber cross sections in the four experimental groups (Control, EMS, Stroke, Stroke + EMS). Diagram shows differences of myofiber cross sectional areas (CSAs) within the four groups. Panel B: Heatmap of relative gene expression differences in the m. quadriceps in response to EMS, Stroke, and Stroke + EMS. Relative expressions of several genes related to heat shock proteins, ER stress, myokines, cytokines, autophagy and sarcopenia were studied in the m. quadriceps of mice using qPCR (*n* = 8–12/group). For EMS vs. Control, Stroke vs. Control, and Stroke + EMS vs. control comparisons, the relative expression of target genes in EMS, Stroke or Stroke + EMS animals was compared to the expression levels detected in Control animals (results are given as a percentage, where Control group = 100%). For the Stroke + EMS vs. Stroke comparison, the relative expression of target genes in Stroke + EMS animals was compared to the expression levels detected in the Stroke group (results are given in percentages, where the Stroke group = 100%). Green labeled *p*-values indicate significant changes. Abbreviations: RT-qPCR: reverse transcription-quantitative polymerase chain reaction; ER stress: endoplasmic reticulum stress. Data are given as mean ± SD. Non-normal distribution data was analyzed by using Kruskal-Wallis One-Way ANOVA on ranks (A) or the non-parametric Mann-Whitney *U* test was used for pairwise comparisons (B), while for normal distribution data (Shapiro-Wilk normality test, *p* = 0.654) the parametric Student’s t-test was performed for pairwise comparisons (B), *p* < 0.05∗.

**Fig. 5:**
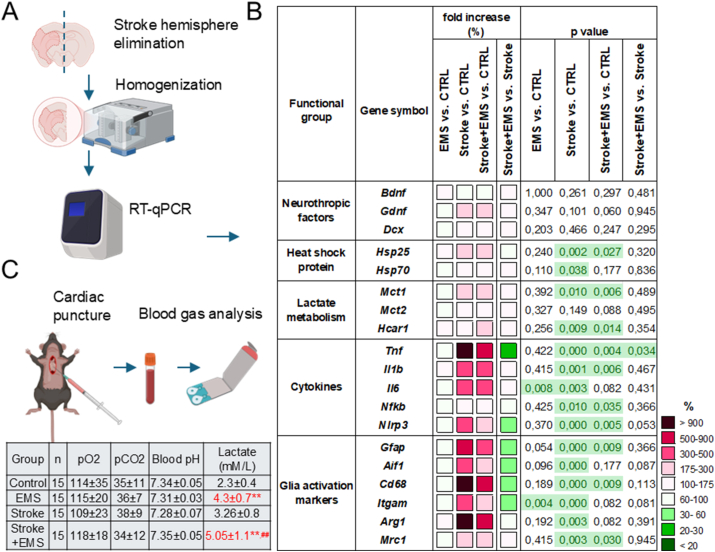
Analysis of gene expression in brain samples, and blood lactate levels 3 days after stroke. Panel A: Cartoons show the processing of brain samples indicated by blue arrows: brain removal and elimination of the left, ipsilateral (stroke) hemisphere, homogenization and RT-qPCR analysis. Panel B: Heatmap of relative gene expression differences in the brain in response to EMS, Stroke, and Stroke + EMS. Relative expressions of several genes related to neurotrophic factors, heat shock proteins, lactate metabolism, cytokines and glia activation markers were studied in the stroke hemisphere of mice using qPCR (*n* = 24). For EMS vs. Control, Stroke vs. Control, and Stroke + EMS vs. control comparisons, the relative expression of target genes in EMS, Stroke or Stroke + EMS animals was compared to the expression levels detected in Control animals (results are given as a percentage, where Control group = 100%). For the Stroke + EMS vs. Stroke comparison, the relative expression of target genes in Stroke + EMS animals was compared to the expression levels detected in the Stroke group (results are given in percentages, where the Stroke group = 100%). Green labeled *p*-values indicate significant changes. Panel C: Schematic cartoons display cardiac puncture, blood sampling and microfluidic analysis. The table shows blood lactate levels in line with blood gases. Red values indicate significant differences. Data are given as mean ± SD. The distribution of data was evaluated by a Shapiro-Wilk test of normality (B: *p* = 0.712, C: *p* = 0.647), followed by a One-Way ANOVA with Holm-Sidak post-hoc test (C), or a Student’s t-test for pairwise comparisons (B), *p* < 0.01∗∗; ∗∗: vs. Control, ##: vs. Stroke.

## Results

### EMS attenuates infarct size and improves neurological outcomes after stroke

The mortality rate in our AIS model was 28%, which is consistent with previous literature reporting rates of up to 50% following MCAO in mice [19]. Sixteen of the 58 animals did not survive to Day 1 and were excluded from the analysis. Infarct size was assessed after three consecutive days of EMS treatment using TTC and hematoxylin–eosin (HE) staining (Fig. 2). TTC-stained sections showed significantly reduced infarct size in the Stroke + EMS group compared with Stroke controls (30.41 ± 9.22 vs. 45.24 ± 8.92% of the affected hemisphere; Stroke + EMS vs. Stroke) (Fig. 2A&C). Consistently, HE-stained sections revealed higher cell densities in the cortex of Stroke + EMS group (263 ± 76 vs. 178 ± 65 cell count/1000 μm^2^; Stroke + EMS vs. Stroke) (Fig. 2B), indicating reduced neuronal loss. While extensive non-viable tissue (infarcted tissue) was evident in the ipsilateral cortex of Stroke animals (Fig. 2B1), cell viability in Stroke + EMS mice was more comparable to the contralateral hemisphere, supporting a neuroprotective effect of EMS (263 ± 76 vs. 322 ± 76 vs. 298 ± 59 cell count/1000 μm^2^; Stroke + EMS ipsi vs. Stroke + EMS contra vs. Stroke contra) (Fig. 2D).

The neurological deficit was assessed daily for four consecutive days, starting on Day 0 (Fig. 3), using the GNS scale as previously described [15]. Among the seven individual tasks of the GNS, EMS-treated stroke mice showed improvements in spontaneous activity (3 ± 1 vs. 2 ± 1 points on D3; Stroke + EMS vs. Stroke) (Fig. 3A) and in lateral turning when held by the tail (3 ± 1 vs. 2 ± 1 points on D3; Stroke + EMS vs. Stroke) (Fig. 3B). Overall recovery was significantly enhanced, as indicated by a main effect of therapy in the EMS stroke group (14 ± 2 vs. 12 ± 3 GNS points; Stroke + EMS vs. Stroke) (Fig. 3C).

### EMS prevents myofibers and dampens cellular stress in the musculus quadriceps after stroke

The cross-sectional area of myofibers was assessed on 10-μm-thick H&E-stained cryosections obtained from both right and left musculus quadriceps. Although the soleus and gastrocnemius muscles were also collected, the final analysis focused on the m. quadriceps, as this muscle exhibited the most pronounced EMS-induced gene expression changes (Suppl. Fig. 1). The cross-sectional area of quadriceps myofibers was significantly reduced in the stroke group (955.4 ± 730 vs. 1618.7 ± 853.9 μm^2^; Stroke vs. Control), indicating muscle wasting, whereas no reduction was observed in EMS-treated mice (1497.6 ± 668.9 μm^2^; EMS) (Fig. 4A). Notably, EMS restored myofiber size in the Stroke + EMS group to control levels (1529.73 ± 1003.15 vs. 1618.7 ± 853.9 μm^2^; Stroke + EMS vs. Control), demonstrating effective muscle regeneration (Fig. 4A).

Next, we aimed to identify the genetic background underlying the protective effects of EMS in the muscle (Fig. 4B). Therefore, we performed multiple RT-qPCR analyses on the left (ipsilateral) m. quadriceps homogenates to monitor changes in cellular stress markers in response to stroke or EMS. We found marked increases in the mRNA levels of heat shock proteins *Hsp70* (409%, *p* = 0.051), *Hsp25* (372%, *p* < 0.01), and *Hspb8* (170%, *p* < 0.01) in the stroke group compared to controls. However, EMS therapy diminished the expression of these genes, as demonstrated by their significant reduction in the Stroke + EMS group compared with Stroke animals (*Hsp25*: 40%, *p* < 0.05 *Hspb8*: 64%, *p* < 0.05). ER-stress markers *Xbps1* and *Atf4* also showed remarkable increased expression in the Stroke group compared to controls (367%, *p* < 0.01 and 284%, *p* < 0.001, respectively). On the other hand, we detected significant decrease in *Atf4* (63%, *p* < 0.05) and *Chop* expression (58 %, *p* < 0.5) in the Stroke + EMS group compared to Stroke animals (Fig. 4B).

The expression of *Erfe*, encoding the myokine myonectin, was significantly induced by AIS (246%, *p* < 0.05) but was not substantially affected by EMS therapy. Two additional myokine-related transcripts, *Fndc5* (encoding irisin) and *Ctsb* (encoding cathepsin B), were also examined because of their reported neuroprotective effects [20]; however, no significant changes in their expression were observed (Fig. 4B).

We also examined the gene expression of several cytokines in our experimental setting. The *Il6* and *Il1b* gene mRNA levels were elevated in the muscle tissue of Stroke animals compared with Controls (180%, *p* = 0.055 and 239%, *p* < 0.05, respectively). While *Il1b* expression showed further elevation by EMS (373% of control, *p* < 0.01), *Il6* levels decreased in Stroke + EMS animals (122%). Moreover, the *Tnf* (gene coding for TNF-α) expression showed an increasing trend in the Stroke + EMS group compared with Controls (225%, *p* = 0.058). Notably, the expression levels of the anti-inflammatory cytokines *Il10* and *Tgfb* were significantly elevated by EMS therapy in both healthy (242%, *p* < 0.05 and 140%, *p* < 0.05, respectively) and in disease model animals (264%, *p* < 0.01 and 144%, *p* = 0.05, respectively; Stroke + EMS vs.Stroke) (Fig. 4B).

Among autophagy markers, *P62* and *Lc3* mRNA levels increased significantly in the Stroke group (278%, *p* < 0.05 and 166%, *p* < 0.01, respectively), while, interestingly, *P62* expression was attenuated in the Stroke + EMS group (49%, *p* = 0.051 compared to Stroke group) (Fig. 4B).

Finally, we found a significant reduction in the sarcopenia marker, *Myh8* gene expression, in response to EMS in the muscle of healthy animals (57%, *p* < 0.01) (Fig. 4B).

### EMS diminishes inflammatory gene expression in the brain after stroke

To discover the molecular mechanisms underlying the neuroprotective effects of EMS therapy in the brain, we analyzed the relative expression of genes involved in cellular stress regulation, lactate metabolism, inflammation, and glial activation (Fig. 5A&B). Brain homogenates from the left (ipsilateral, stroke) hemisphere were used for RT-qPCR analyses. To assess the effects of EMS therapy, ischemic stroke, and their combination, we compared the values obtained for the EMS, Stroke, and Stroke + EMS groups with those of the control group (set to 100% for all comparisons). Additionally, we performed a direct comparison of Stroke + EMS and Stroke groups, to better visualize the therapeutic effects in the disease model. For this relation the values for the Stroke animals were considered 100% (Fig. 5B).

A more than twofold increase in the mRNA level of *Hsp25* was observed in Stroke animals compared with Controls (263%, *p* < 0.01), while *Hsp70* showed only a slight elevation (136%, *p* < 0.05). EMS had no significant effect on the expression of these stress-response genes (Fig. 5B).

Since muscle derived lactate (see below) might serve as an alternative energy source for the brain under metabolic stress, we aimed to investigate the genes involved in lactate metabolism. The Monocarboxylate transporter-1 (*Mct1*) and the Hydroxycarboxylic acid receptor 1 (*Hcar1*) showed a modest but significant increase in the Stroke and Stroke + EMS groups compared with Controls (179%, *p* < 0.05 and 164%, *p* < 0.01, respectively). The *Mct1* and *Hcar1* mRNA elevation might be mechanistically linked to the elevated blood lactate levels (Fig. 5B) and we further explain this possible signaling in the discussion.

As expected, inflammatory gene levels increased significantly in the left hemispheres three days after stroke. *Il1b* and *Il6* expression rose approximately fourfold in response to Stroke (464%, *p* < 0.01 and 396%, *p* < 0.01, respectively). Although these genes were not notably influenced in the Stroke + EMS group, *Il6* expression showed a slight but significant decrease in the EMS group compared to controls (71%, *p* < 0.01). A similar fourfold increase was observed for *Nlrp3* (452%, *p* < 0.001), while *Tnf* levels rose dramatically in Stroke animals compared with Controls (2419%, *p* < 0.001). Notably, EMS treatment substantially reduced the expression of these markers (to 25%, *p* < 0.05 and 57%, *p* = 0.053, respectively; Stroke + EMS vs. Stroke).

A similar trend was observed for glial activation marker genes. All examined astrocyte and microglial markers were significantly elevated in Stroke animals compared with Controls (*Gfap*: 883%, *p* < 0.001; *Aif1*: 437%, *p* < 0.001; *Cd68*: 1152%, *p* < 0.001; *Itgam*: 391%, *p* < 0.001; *Arg1*: 1067%, *p* < 0.01; *Mrc1*: 200%, *p* < 0.01). Importantly, EMS therapy reduced these values by nearly half, as seen for the astrogliosis marker Gfap (55%) and the microglial markers Aif1 (53%), Cd68 (48%), and Itgam (57%) in the EMS + Stroke group compared to Stroke animals. Although the differences are not statistically significant, the decreasing pattern of the inflammation-related glial markers, reinforces the potential neuroprotective effects of the EMS therapy (Fig. 5B). In contrast, the expression of the anti-inflammatory M2 microglial markers Arg1 and Mrc1 were not substantially reduced in the Stroke + EMS group (77% and 107% respectively, compared with Stroke group) (Fig. 5B).

### EMS elevates blood lactate levels

The measured blood gas parameters: including the O_2_, CO_2_, and pH levels remained within physiological ranges, but lactate showed an individual tendency that was significant both in the EMS and Stroke + EMS groups (5.18 ± 1.1 vs. 4.4 ± 0.8 vs. 3.26 ± 0.7 vs. 2.27 ± 0.4; Stroke + EMS vs. EMS vs. Stroke vs. Control) (Fig. 5C). It is important to note that stroke alone was associated with a tendentious elevation in blood lactate levels (*p* = 0.058 vs. Control), which was further, significantly augmented by EMS in both the EMS (*p* < 0.001 vs. Control) and Stroke + EMS (*p* < 0.001 vs. Control) groups (Fig. 5C). Although these profound elevations in the lactate concentration were measured 3 days after stroke and after the last EMS sessions each (median time after EMS = 20 min), we propose an established lactate-mediated, neuroprotective signaling to the brain that may underlie the elevated monocarboxylate transporter (*Mct1*), lactate receptor (*Hcar1*), and anti-inflammatory mRNA levels observed above (Fig. 5B) and discussed below.

## Discussion

This study demonstrates that electrical muscle stimulation (EMS) exerts novel, significant neuroprotective effects after stroke by reducing neuroinflammation and infarct size while improving the neurological deficit in mice. Neuroprotection was achieved here by the constant current, 4 Hz frequency stimulation of both lower limbs of anesthetized mice for consecutive 3 days after stroke. The applied B-SES paradigm was previously established [18] but has never been tested in cerebrovascular disease models. Our results confirm the novel and complex peripheral (muscle) and central (brain) gene expression changes in response to EMS, that highlight the mechanisms behind the neuroprotective effects of EMS. Our EMS protocol was applied in a mouse reperfusion model of ischemic stroke, thereby increasing the translational potential of this work in stroke care, especially for patients who achieve successful recanalization. Based on these results we propose EMS therapy as adjuncts to conventional rehabilitation after stroke to achieve neuroprotection in patients with limited voluntary movement.

In fact, early mobilization is an established component of acute stroke care and is associated with reduced complications of immobility and improved functional outcomes when applied a personalized manner [21]. Randomized studies further indicate that appropriate protocol for early mobilization improves functional capacity, enhances activities of daily living, and may shorten hospital length of stay [8,22]. In patients unable to mobilize voluntarily, EMS may be complementary strategy that reproduces key biological effects of early physical activity. Systematic reviews and meta-analyses confirm that EMS can lead to significant improvements in activities of daily living and functional outcomes, especially when applied to the upper limb during the early or subacute post-stroke period [[23], [24], [25]]. Beyond functional effects, EMS may positively influence post-stroke pathologies through exercise-mimetic anti-inflammatory mechanisms.

One important factor that further necessitates individualized application of EMS is the timing of intervention. Clinical evidence suggests that very early rehabilitation or sensory stimulation after ischemic stroke may be detrimental [26]. Experimental data also support this possibility [27]. Taken together, these findings indicate that the translational implications of our EMS protocol require further investigation and additional data.

### Protective role of EMS in the muscle

Based on our results, we propose that the observed neuroprotection—reflected by (i) reduced infarct size on TTC staining and preserved cellular viability on H&E staining, and (ii) enhanced spontaneous activity and attenuated neurological deficits—originates from the stimulated skeletal muscle. We selected the ipsilateral (left) musculus quadriceps to minimize potential confounding effects of stroke-induced paralysis and denervation on cytokine/myokine release following EMS, thereby isolating the direct impact of EMS on gene expression. Notably, our pilot morphological analyses (H&E staining) revealed no significant differences between ipsilateral and contralateral m. quadriceps at this stage (3 days post stroke), suggesting limited structural impact on the measured outcomes. Nevertheless, this approach may influence interpretation, as contralateral (paralyzed) muscles could exhibit distinct, lower cytokine responses, which warrants further investigation.

In the musculus quadriceps, stroke led to pronounced muscle fiber atrophy, upregulation of cellular stress markers, and activation of inflammatory and autophagic pathways— all hallmarks of post-stroke muscle wasting [28]. EMS treatment effectively prevented the reduction in myofiber size and attenuated the expression of stress-related genes such as *Hsp25, Hspb8*, and *Atf4,* suggesting a decrease in proteotoxic stress and improved cellular viability. In line with our findings, earlier studies have also reported that low frequency muscle stimulation might (i) preserve muscle mass and function but also [29,30] (ii) might ease the proteotoxic stress by activating protective pathways and suppressing stress-related catabolic signals [29,30]. The normalization of *P62* expression by EMS in our study supports a balanced autophagic response, consistent with preserved muscle integrity. In concert, others have demonstrated an enhanced satellite cell fusion after EMS, that improves autophagy-related signaling through AMPK, Akt, and PGC-1α, and even protects against lipid-induced ER stress, all of which contribute to maintaining proteostasis [29,31].

Inflammatory cytokine expression showed a more anti-inflammatory profile in EMS-treated animals. In contrast, stroke resulted in a marked upregulation of *Il1b* and *Tnf*, while *Il6* levels showed only partial normalization. Importantly, EMS elevated anti-inflammatory cytokines *Il10* and *Tgfb* in both healthy and post-stroke conditions, indicating that EMS promotes a systemic anti-inflammatory milieu. The reduction in *Myh8* expression in healthy muscles further suggests that EMS helps prevent early sarcopenic changes linked to disuse muscle atrophy. These results also stand in line with very recent findings, where EMS was found to be anti-inflammatory both in experimental model and in a clinical setting [32,33].

### Elevated lactate level in the blood in response to EMS

Our results showed that blood lactate levels elevated significantly in response to EMS. Muscle contraction is a major source of systemic lactate production. During repeated or sustained muscle activation, glycolytic flux increases, leading to elevated lactate release from skeletal muscle into the blood [34]. We demonstrate here that this mechanism is not only limited to voluntary exercise but can also be triggered by electrical muscle stimulation (EMS). In fact, the resulting rise in blood lactate might provide an efficient and rapidly available energy source that can be taken up by the brain and utilized by neurons under metabolic stress, such as stroke [35]. We propose that the significantly increased expression of the monocarboxylate transporter 1 (*Mct1*) and the lactate receptor *Hcar1* mRNAs in brain samples from the Stroke and Stroke + EMS groups reflects the establishment of a blood-to-brain lactate gradient, which may have been a key driver of neuroprotection in our study. In agreement, stroke-induced upregulation of *Mct1* and *Hcar1*, which occurs independently of EMS, may enable the brain to more effectively utilize EMS-induced increases in circulating lactate by facilitating its transport into the brain along a favorable concentration gradient. The elevated *Mct1/Hcar1* expression observed after stroke likely represents a compensatory mechanism to support cerebral energy metabolism [36,37], but its functional impact is maximized when accompanied by the heightened systemic lactate levels elicited by EMS in our model. Muscle-derived lactate can enter the brain and activate HCAR1 receptors on microglia and endothelial cells, suppressing NF-κB signaling and thereby reducing *Tnf* production as seen in our model. Lactate also enhances cellular energy metabolism and promotes an anti-inflammatory microglial phenotype, which might have further contributed to the lower *Tnf* in the Stroke + EMS group. We therefore further hypothesize that the EMS-induced rise in circulating lactate in the Stroke + EMS group provides an additional metabolic substrate, thereby augmenting the endogenous neuroprotective response. Our findings are further supported by an ongoing clinical study investigating whether lactate administration can serve as a secondary energy substrate following ischemic stroke (https://clinicaltrials.gov/study/NCT04858139). Taken together, these findings suggest a potential neuroprotective role for muscle-derived lactate after stroke, warranting further experimental investigation.

### Neuroprotective role of EMS in the brain

Our study provides direct evidence to confirm that improved muscle state could contribute to neuroprotection in the brain after a cerebrovascular disease state. Our results demonstrate for the first time that, in the brain, EMS treatment blunted the stroke-induced overexpression of proinflammatory and glial activation markers, including *Il1b, Il6, Nlrp3, Tnf, Gfap, Aif1*, and *Cd68*. This pattern points to attenuated neuroinflammation and reduced activation of astrocytes and microglia [38,39]. Importantly, neuroinflammation is a known mediator of infarct maturation [40,41], therefore this anti-inflammatory effect of EMS might also explain the reduction of infarct sizes in the Stroke + EMS group. These effects of EMS might be attributed to the muscle derived production of anti-inflammatory molecules, “myokines” that could enter the brain. Although we did not find any significant movement of the widely recognized myokine mRNA levels (irisin, cathepsin-B, myonectin), we propose the effects of lactate as an emerging myokine [35]. Lactate signaling via the HCAR1 (GPR81) receptor suppresses pro-inflammatory pathways, reduces the release of cytokines such as TNF-α and IL-1β from microglia [42,43]. Experimental evidence indicates that elevated lactate levels attenuate microglial activation, limit inflammasome signaling, and preserve neurovascular integrity after ischemic injury, thereby contributing to improved tissue outcome [44]. Interestingly, the expression of anti-inflammatory M2 microglial markers (Arg1 and Mrc1) remained relatively stable, implying that EMS may suppress detrimental M1-type activation while maintaining protective M2 responses. Together, these molecular changes provide mechanistic support for the histological and behavioral improvements observed here.

Collectively, using a mouse reperfusion model of stroke, we demonstrate that EMS shows translational potential and may represent a therapeutic option following complete recanalization in patients with acute ischemic stroke. Our findings suggest that EMS confers neuroprotection after stroke through dual central and peripheral anti-inflammatory mechanisms. Peripherally, it preserves skeletal muscle structure and limits systemic inflammation; centrally, it reduces neuroinflammatory signaling and glial activation in the ischemic hemisphere. Above the muscle-derived humoral signaling, these effects of EMS may also stem from the activation of afferent neural pathways that link peripheral muscle activity to central neuronal homeostasis, as previously suggested in studies of exercise-induced neuroprotection [45,46]. Preclinical studies demonstrate that post-stroke sensory stimulation can exert neuroprotective effects by modulating cortical excitability, enhancing cerebral perfusion, and engaging activity-dependent plasticity mechanisms [47,48]. Importantly, human studies further show that somatosensory stimulation delivered during the subacute and chronic post-stroke phases enhances cortical excitability and functional recovery [49,50]. Although neuroplasticity may have been facilitated by peripheral EMS in our cohort, the specific contribution of sensory pathway activation was not directly investigated in this study. Furthermore, extending the follow-up period in our model is warranted, as a 3-day survival window provides limited insight into longer-term functional or histopathological outcomes. Longer observational intervals will be essential to more accurately characterize the durability and full extent of EMS-mediated neuroprotection. The successful translation of EMS will require personalized follow-up strategies, particularly with respect to treatment timing, to avoid potential adverse effects associated with overly early mobilization after stroke [26]. Taken together, EMS emerges as a promising adjunctive therapy that bridges peripheral stimulation with central recovery and may offer a safe and practical approach to improving post-stroke outcomes.

## Author contribution statement

A.T.: Methodology, investigation.

P. K.: Methodology, investigation.

H.U.: Methodology.

R.H.: Methodology.

Zs. R.: Methodology, investigation.

E. G.: Methodology.

F. B.: Writing - Review & Editing.

Zs. T.: Data curation, Writing - Review & Editing.

E. F.: Data curation, Writing - Review & Editing, Funding acquisition.

M. E. T.: Investigation, Supervision, Data curation, Writing – Review & Editing, Funding acquisition.

Á. M.: Methodology, Investigation, Supervision, Project administration, Writing - Original Draft, Visualization, Funding acquisition.

## Declaration of competing interest

The authors declare that they have no known competing financial interests or personal relationships that could have appeared to influence the work reported in this paper.
