# Supplementary material for: Electric muscle stimulation attenuates neuroinflammation and improves the outcome of acute ischemic stroke in mice
Source: Neurotherapeutics. 2026 Jun 2;23(4):e00933. doi: 10.1016/j.neurot.2026.e00933 (PMC13254675; doi:10.1016/j.neurot.2026.e00933)

Representative mRNA expression elevations (Erfe and Hsp25) in the musculus quadriceps, musculus gastrocnemius, and musculus soleus

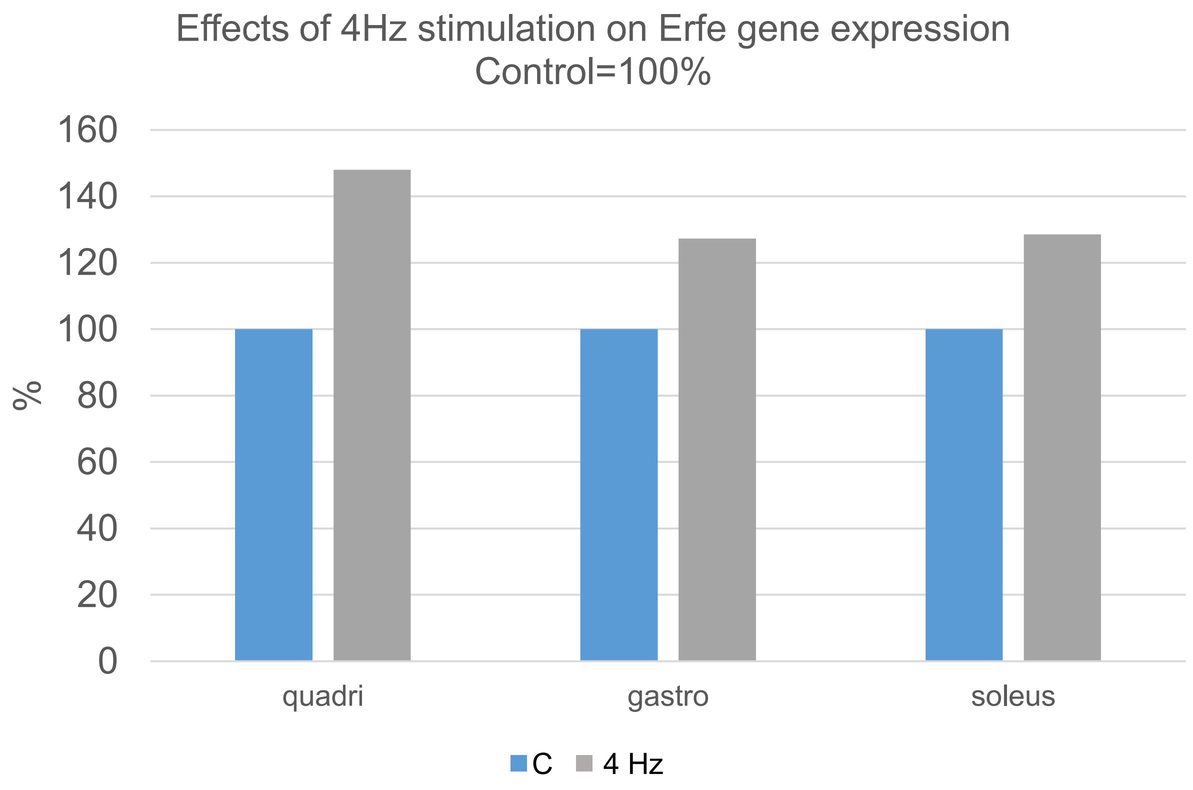

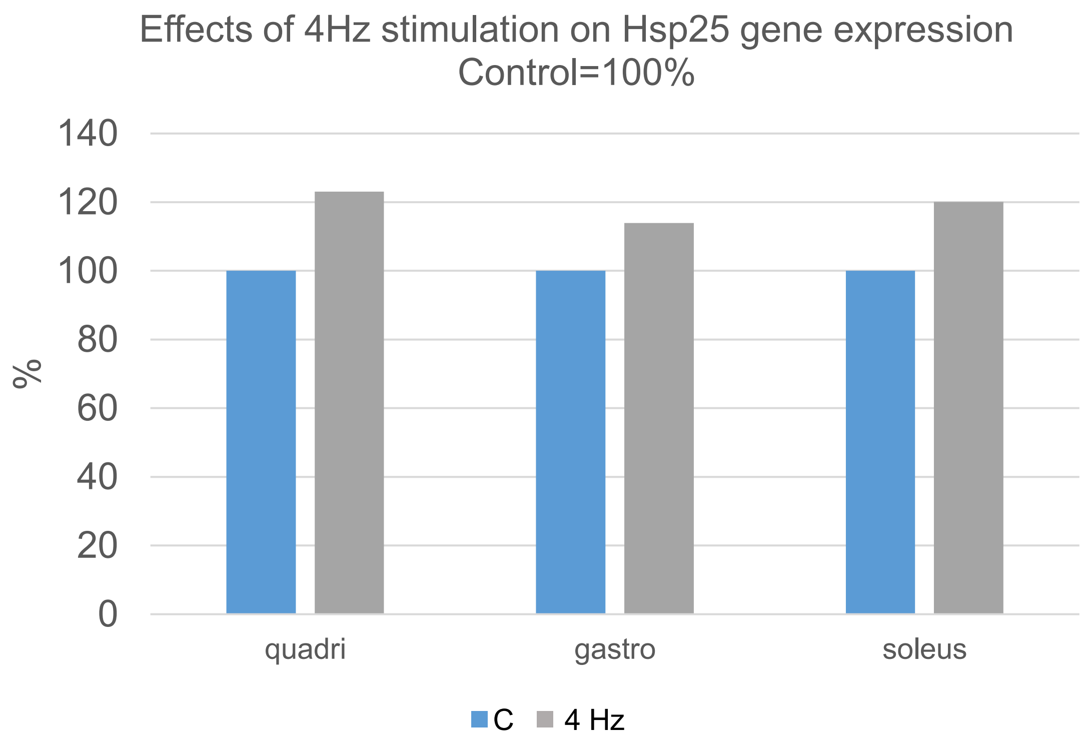

Supplement: Multimedia component 1 [file mmc1.pdf]
